# Volatolomics of Three South African *Helichrysum* Species Grown in Pot under Protected Environment

**DOI:** 10.3390/molecules26237283

**Published:** 2021-11-30

**Authors:** Basma Najar, Ylenia Pieracci, Claudio Cervelli, Guido Flamini, Luisa Pistelli

**Affiliations:** 1 Dipartimento di Farmacia, Università di Pisa, Via Bonanno 6, 56126 Pisa, Italy; basmanajar@hotmail.fr (B.N.); luisa.pistelli@unipi.it (L.P.); 2 Centro di Ricerca Orticoltura e Florovivaismo (CREA-OF), Corso Inglesi 508, 18038 Sanremo, Italy; claudio.cervelli@crea.gov.it; 3 Centro Interdipartimentale di Ricerca Nutraceutica e Alimentazione per la Salute “Nutrafood”, (NUTRAFOOD), Università di Pisa, Via del Borghetto 80, 56124 Pisa, Italy

**Keywords:** *Helichrysum decorum*, *Helichrysum lepidissimum*, *Helichrysum umbraculigerum*, sesquiterpenes, hydrodistillation, SPME, GC-MS

## Abstract

*Helichrysum decorum* DC, *Helichrysum lepidissimum* S. Moore, and *Helichrysum umbraculigerum* are three species traditionally used in the South African medicine. The present work deals with the investigation of the spontaneous emission and the essential oils obtained from these plants cultivated in open field under uniform conditions. Fractions of the volatile organic compounds of the three species were rich in monoterpene hydrocarbons, representing more than 70% of the total composition. Pinene isomers were the most representative compounds: β-pinene in *H. decorum* (53.0%), and α-pinene in *H. lepidissimum* (67.9%) and *H. umbraculigerum* (54.8%). These latter two species evidenced an important amount of sesquiterpene hydrocarbons (SH) especially represented by γ-curcumene (*H. lepidissimum*) and α- and β-selinene (*H. umbraculigerum*). On the contrary, in the EOs, sesquiterpenes compounds prevailed, representing more than 64% of the identified fraction to reach more than 82 and 87% in *H. umbraculigerum* and *H. lepidissimum,* respectively. Although the chemical classes and their relative abundances were comparable among the three species, the individual compounds of EOs showed large differences. In fact, caryophyllene oxide (26.7%) and γ-curcumene (17.4%) were the main constituents in *H. decorum*, and *H. lepidissimum* respectively, while *neo*-intermedeol (11.2%) and viridiflorol (10.6%) characterized *H. umbraculigerum*.

## 1. Introduction

The genus *Helichrysum*, belonging to the family Asteraceae, comprises more than 500 species, of which almost half are indigenous to South Africa [1,2,3]. Different species of *Helichrysum* are widely used in the traditional local medicine, thanks to the variety of secondary metabolites that the plants belonging to this genus can produce [3]. Their aerial parts are employed as herbal teas for the treatment of respiratory issues, digestive problems, as diuretic and anti-inflammatory agents, and for other purposes [4,5]. Several *Helichrysum* species are appreciated for their aroma profile, strictly connected to the presence of essential oils, produced and stored in the glandular trichomes located in almost all the vegetative epigeal parts of the plant [4]. The essential oil plays an important role for the taxonomic attribution of these species [6], as well as for their biological activities [7]. *Helichrysum* species are, indeed, characterized by a huge genetic variability, due to their polymorphisms, as a consequence of different environmental and growing conditions. It was observed that both the different morphological characters of the plants and the chemotypes of their EOs are attributable to the genetic heritage as well, and therefore the chemical composition can be used for the taxonomic identification [8]. In the last decades, it is no coincidence that the essential oils obtained from different species of this genus received increasing interest, for both their chemical composition and their biological activities [2,7,9].

Continuing our research on the utilization of *Helichrysum* spp. indigenous of South Africa, in collaboration with Centro di Ricerca Orticoltura e Florovivaismo (CREA-OF) located in Sanremo (Italy), three new *Helichrysum* species were investigated. *Helichrysum lepidissimum* S. Moore and *Helichrysum umbraculigerum* Less. are biennial or perennial herb shrubs, *Helichrysum decorum* DC is a plant growing in sandy grassland or open woodland from sea level to 900 m. The African Zulu inzagomas (diviners) smoked/inhaled or burned unspecific parts of the plant, which resulted in a trance state [3]. Growing on rocky grounds in submontane areas, *H. lepidissimum* is a perennial shrub [10] from which Mkhize (2015) isolated lepidissipyrone [11]. This compound showed a structure similar to arzanol, isolated from *H. italicum* ssp. *microphyllum*, and it was known for its antioxidant, anti-inflammatory and anti-HIV activities. According to Lourens et al., 2008 the powder and ointment prepared from this species are used as a body ointment in traditional usage [3]. *H. umbraculigerum,* instead, is a perennial erected plant reported in several studies as the main natural source of cannabigerol [12].

Despite their important traditional uses, investigations on these *Helichrysum* species are lacking, and the studies reported in the literature only cite them without any other research on their biological activity or on their secondary metabolites content. This work aims to evaluate the chemical composition of both the spontaneous volatile emissions and the essential oils of the three South African species of *Helichrysum* cultivated at the CREA-OF (Italy). To the best of our knowledge these investigations have never been previously reported in the literature.

## 2. Results and Discussions

### 2.1. Volatiles Organic Compounds (VOCs)

Thirty-six compounds were detected by GC-MS methods in the spontaneous volatile emissions, with a percentage of identification ranging between 99.5% to 100% of the whole volatilome (Table 1). *H. umbraculigerum* was the richest plant for variety of compounds emitted (21) compared to *H. decorum* (16) and *H. lepidissimum* (15). Interestingly, only four constituents were shared by the samples, and two of them (β-pinene and α-pinene) were major ones.

The main class of constituents for the three species was represented by monoterpene hydrocarbons, with 93.3% in *H. decorum*, followed by *H. umbraculigerum* (75.2%) > *H. lepidissimum* (70.3%). β-Pinene (53.0%) dominated the composition of *H. decorum* together with sabinene (23.8%). These two compounds, along with α-pinene (11.6%), represented more than 88% of the volatile fraction. Similar amounts of β-pinene (55.2%) were also found by Bandeira Riedel [1] analyzing the spontaneous emission of the flower of *H. arenarum* (L.) Moench. Sabinene instead, was the major constituent of *H. petiolare* Hilliard & B.L. Burtt (about 50% of the whole HS composition) [13], *H. cooperi* (34.4%), and *H. edwardsii* (29.2%) [7]. VOCs of *H. lepidissimum* and *H. umbraculigerum* were richer in the α-isomer of pinene (67.9% and 54.8%, respectively), compared that of to β-pinene (1.1% and 2.7%, respectively), as also observed in the previously mentioned species. The predominance of α-pinene was also observed for *H. pandurifolium* (25.7%) and *H. trilineatum* (64.8%), previously investigated by our research team [7].

Substantial amounts of sesquiterpene hydrocarbons (SH) also characterized *H. lepidissimum* and *H. umbraculigerum*, representing 28.0% and 21.9% of the spontaneous emissions, respectively. Nevertheless, they showed quite different main sesquiterpene compounds. In fact, γ-curcumene (23.1%) was the major SH detected in *H. lepidissimun*, while *H. umbraculigerum* was characterized by higher percentage of the two isomers of selinene: α (6.2%) and β (3.9%), together with α-humulene (4.6%). γ-Curcumene (10.75) and β- (8.5%) and α-selinene (7.3%) were also the main compounds found in the spontaneous emission of *H. araxinum* Takht. ex Kirp, which in fact had SH as the most representative chemical class (79.5%) [2].

### 2.2. Essential Oil Chemical Composition and Yield

The complete chemical composition and the hydrodistillation yields of the essential oils (EOs) obtained from the dried aerial parts of *H. decorum*, *H. lepidissimum* and *H. umbraculigerum* are reported in Table 2. Altogether 112 compounds were identified, representing 92.5 to 97.1% of the total chemical composition. *H. umbraculigerum* presented the highest number of constituents (47 vs. 41 in both *H. decorum* and *H. lepidissimum*), as in VOC analysis. Remarkable was the fact that these oils, apart from 3 minor constituents, had no compounds in common. Moreover, the essential oil yield of *H. lepidissimum* was 0.6% *w*/*w*, while for the other two species it was so low that it could not be determined. In general, the species of this genus are known to produce low amounts of essential oil [7].

Concerning the chemical composition, sesquiterpenes were the most represented class of compounds in all the EOs. The oxygenated form prevailed, accounting for 48.5, 60.4 and 55.2% in *H. decorum*, *H*. *lepidissimum* and *H. umbraculigerum*, respectively, while the hydrocarbon form ranged from 17.5% in *H. decorum* to 26.5 and 26.9% in *H.* *lepidissimum* and *H. umbraculigerum*, respectively. These three EO samples showed great differences in their compositions. Caryophyllene oxide was the main constituent in *H. decorum* (26.7%), followed by β-caryophyllene (8.4%). Despite the oxygenated sesquiterpenes (OS) dominated the *H. lepidissimum* EO, γ-curcumene, a sesquiterpene hydrocarbons, was the main constituent of this oil (17.4%), followed by β-bisabolol (12.5%), *epi*-globulol (7.4%), and rosifoliol (7.2%). The *H. umbraculigerum* essential oil instead was characterized by a predominance of OS, i.e., *neo*-intermedeol (11.2%) and viridiflorol (10.6%), followed by SH α-selinene (9.2%) and β-selinene (6.2%).

The presence of high percentages of caryophyllene oxide is not very common in the genus *Helichrysum,* even though this compound was reported by Rabehaja, D.J.R. et al. for the Malagasy *H. benthamii* Viguier & Humbert (4.0%) [17]. These authors also evidenced a similar behaviour with the same species concerning the predominance of sesquiterpenes, with prevalence of the oxygenated ones (73.5%) in *H. hirtum* Humbert. Caryophyllene oxide was also detected in other South African species, such as *H. cymosum* (L.) D. Don subsp. *cymosum* studied by Giovanelli et al. [4]. γ-Curcumene, a sesquiterpene hydrocarbon typical of the EO of *H. italicum* (Roth) G. Don was reported as the main component of Serbian samples [18]. This chemical compound, together with rosifoliol, was detected in appreciable percentages in the EO of *H*. *italicum* Don. subsp. *microphyllum* (Willd.) Cambess, also known as *H*. *italicum* subsp. *tyrrhenicum*, which is widely employed in aromatherapy [19]. Aćimović at al. [18], in fact, evidenced that *Helichrysum* chemotypes containing γ-curcumene as main component could be used in perfumery industries thanks to their appreciated fragrances, as well as in food and pharmaceutical industries as natural preservatives.

*H. foetidum* (L.) Cass., *H. montanum* DC. [5], and *H. excisum* (Thunb.) Less. [20] were characterised by the presence of an amount of viridiflorol comparable to that of *H. umbraculigerum* (ranged between 10–20%). On the contrary, *H. pandurifolium* Schrank had nearly six times the amount of viridiflorol found here (60% of the whole EO composition) [7]. *Neo*-intermedeol was identified only in *H. araxinum* [2] and in *H. italicum* subsp. *italicum* from Montenegro [21], but with content substantially lower than in the sample analyzed herein. Remarkable quantities of β-selinene were also identified in the EOs of *H. archimedeum* C. Brullo & Brullo [22], *H. hyblaeum* Brullo [22], *H. odoratissimum* (L.) Sweet [7], and *Helichrysum thianschanicum* Regel [23]. α-Selinene, instead, was the major component of the EO of *H. araxinum*, which showed also good amounts of the β-isomer [2]. Fair amounts of both the molecules were also detected in *H. chasmolycicum* P.H.Davis [24]. Interestingly, the high percentage of apocarotenoids (AC) in *H. decorum* (12.5%), mainly represented by hexahydrofarnesylacetone (5.5%), followed by the ionones *trans*-β-ionone (3.8%), dihydropseudoionone (1.4%), *(E,E)*-farnesylacetone (1.0) and dihydro-γ-ionone (0.8%). These latter compounds were completely absent in *H. umbraculigerum,* where a low percentage of AC was observed too (3.2%), and hexahydrofarnesylacetone was almost the unique AC constituent (2.6%). This constituent was also reported in the Turkish species *H. chinophilum* Boiss. & Balansa (3.2%) [8].

Noteworthy is the appreciable percentage of oxygenated diterpenes in the *H. lepidissimum* EO (5.6%) with geranyl linalool as unique identified compound. This class of constituent was also found in other *Helichrysum* species EOs, even though in higher percentages [7,25].

## 3. Materials and Methods

### 3.1. Plant Material

The South African *Helichrysum* plants studied in the present work (see Table 3) belong to the collection of Centro di Ricerca Orticoltura e Florovivaismo (CREA-OF), located in Sanremo, Italy. The seeds were purchased from specialized companies in sailing seeds of African plant species (Silver Hill-PO Box 53108, Kenilworth, 7745 Cape Town, South Africa and B&TWorld Seeds-Paguignan, 34210 Aigues Vives, Gard, France). The plants were grown under the same edaphic substrate (perlite (2:1 *v/v* added with 4 g/L slow-release fertilizer) and climatic conditions (Csa in Köppen-Geiger climate classification with an average annual temperature of 16 °C and an annual rainfall of about 700 mm; frosts are light and very rare). After clonal propagation, the plants grew in pots in the open air and were periodically watered. Flowering took place after one year. A voucher sample of each plant was deposited at the herbarium of the Hanbury Botanical Gardens (La Mortola–Ventimiglia, Imperia, Italy) (Table 3).

### 3.2. Spontaneous Emission Analysis and EO Extraction

Living fresh plant material (almost 1 g) was the subject of the HS-SPME (head space-solid phase microextraction) analyses which was performed using 100 µm polydimethylsiloxanes (PDMS) fiber manufactured by Supelco Ltd. (Bellefonte, PA, USA). As recommended by the manufacturer’s instruction, prior to the analyses, the fiber was conditioned at 250 °C for 30 min in the injector of a gas chromatograph. The plant material was placed in a 50 mL glass vial, covered with an aluminum foil, and then left for 60 min (equilibration time). Exposition of the fiber in the headspace phase of the samples took place for 15 min at a temperature of 23 °C. Subsequently, the fiber was transferred to the injector of the gas chromatograph (temperature 250 °C), where the analytes were thermally desorbed [27]. The composition of the compounds desorbed from SPME fiber was examined using GC-MS.

### 3.3. Essential Oil Hydrodistillation

The essential oil was obtained from the dried aerial parts of the three species of Helichrysum by hydrodistillation with a Clevenger-type apparatus, performed for 2 h at 100 °C, according to the method reported in the European Pharmacopoeia [28]. The hydrodistillation was carried out in triplicates, on 50 g of plant material and the collected essential oil was refrigerated at 4 °C and maintained far from light sources until analyses.

### 3.4. Gas Chromatography—Mass Spectrometry Analyses

The essential oils were diluted to 0.5% in HPLC-grade n-hexane before the injection in the GC–MS apparatus. The GC/EI-MS analyses were performed with an Agilent 7890B gas chromatograph (Agilent Technologies Inc., Santa Clara, CA, USA) equipped with an Agilent HP-5MS capillary column (30 m × 0.25 mm; coating thickness 0.25 μm) and an Agilent 5977B single quadrupole mass detector.

The analytical conditions were set as follows: oven temperature ramp from 60 to 240 °C at 3 °C/min; injector temperature, 220 °C; transfer line temperature, 240 °C; carrier gas helium, 1 mL/min. The injection volume was 1 μL, with a split ratio of 1:25. The acquisition parameters were: full scan; scan range: 30–300 *m*/*z*; scan time: 1.0 s.

The Identification of the constituents was based on a comparison of the retention times with those of the authentic samples, comparing their linear retention indices relative to the series of *n*-hydrocarbons. Computer matching was also used against commercial (NIST 14 and ADAMS 2007) and laboratory-developed mass spectra libraries built up from pure substances and components of commercial essential oils of known composition and MS literature data [14,29,30,31,32,33].

## 4. Conclusions

The present study represents a contribution to increasing the knowledge about the chemical composition of the HSs and the essential oils of three South African *Helichrysum* species that were not studied yet. It should be a starting point for future investigations, which can lead to a more informed employment of these plants, as they are already used in the traditional local medicine. The studied species showed huge differences in the chemical composition of both the spontaneous emissions and the EOs.

The chemical differences of the aroma profile of the studied samples together with their habitus can be exploited for the ornamental use of these plants.

## Figures and Tables

**Table 1:** Complete chemical composition of spontaneous emissions of three analyzed *Helichrysum* samples.

Compounds ^a^	Class	l.r.i ^exp^	l.r.i ^lit^	Relative Abundance (%) ± SD		
				*H. decorum*	*H. lepidissimum*	*H. umbraculigerum*
α-thujene	mh	930	930	0.7 ± 0.08	0.3 ± 0.04	-
α-pinene	mh	939	935	11.6 ± 0.33	67.9 ± 9.09	54.8 ± 5.88
camphene	mh	954	943	0.4 ± 0.13	0.1 ± 0.08	6.4 ± 2.06
β-thujene	mh	966	966	-	-	0.5 ± 0.46
sabinene	mh	975	973	23.8 ± 0.42	0.9 ± 0.11	-
β-pinene	mh	979	974	53.0 ± 0.43	1.1 ± 0.08	2.7 ± 1.29
β-myrcene	mh	991	991	0.6 ± 0.24	-	4.1 ± 0.21
*(Z)*-3-hexenyl acetate	nt	1005	1009	0.4 ± 0.35	-	-
α-terpinene	mh	1017	1018	0.3 ± 0.00	-	0.3 ± 0.25
*p*-methyl anisole	nt	1019	1024	-	0.5 ± 0.16	-
*o*-cymene	mh	1026	1027	0.4 ± 0.05	-	-
limonene	mh	1029	1031	1.8 ± 0.40	-	3.1 ± 0.51
eucalyptol	om	1031	1032	0.9 ± 0.64	1.2 ± 0.27	2.3 ± 1.77
γ-terpinene	mh	1060	1062	0.7 ± 0.05	-	0.8 ± 0.28
terpinolene	mh	1089	1088	-	-	2.5 ± 0.52
linalool	om	1097	1089	-	-	0.6 ± 0.06
β-thujone	om	1114	1115	2.7 ± 0.20	-	-
α-ylangene	sh	1375	1372	-	-	0.5 ± 0.08
α-copaene	sh	1377	1377	1.0 ± 0.04	-	1.4 ± 0.09
isoitalicene	sh	1402	1398	-	0.4 ± 0.22	-
*cis*-α-bergamotene	sh	1413	1415	-	0.4 ± 0.12	-
sesquithujene	sh	1414	1417	-	0.5 ± 0.22	-
β-caryophyllene	sh	1419	1420	0.6 ± 0.18	-	0.9 ± 0.33
α-humulene	sh	1455	1455	-	-	4.6 ± 1.56
sesquisabinene	sh	1464	1461	-	0.9 ± 0.13	-
γ-muurolene	sh	1480	1477	-	-	1.9 ± 0.08
*ar*-curcumene	sh	1481	1483	-	1.6 ± 0.51	-
γ-curcumene	sh	1483	1484	-	23.1 ± 6.31	-
β-selinene	sh	1490	1488	-	-	3.9 ± 0.65
α-selinene	sh	1498	1497	-	-	6.2 ± 0.48
α-muurolene	sh	1500	1499	0.6 ± 0.08	-	-
*epi*-zonarene	sh	1501	1500	-	-	0.9 ± 0.10
β-bisabolene	sh	1506	1509	-	0.3 ± 0.11	-
*trans*-γ-cadinene	sh	1514	1514	-	-	0.2 ± 0.13
β-curcumene	sh	1516	1517	-	0.8 ± 0.37	-
δ-cadinene	sh	1523	1524	-	-	1.4 ± 0.04
Chemical Classes				*H. decorum*	*H. lepidissimum*	*H. umbraculigerum*
Monoterpene Hydrocarbons (mh)				93.3 ± 1.17	70.3 ± 9.43	75.2 ± 5.35
Oxygenated Monoterpenes (om)				3.6 ± 0.20	1.2 ± 0.27	2.9 ± 1.82
Sesquiterpene Hydrocarbons (sh)				2.2 ± 0.30	28.0 ± 7.99	21.9 ± 3.19
Other non-terpene derivatives (nt)				0.4 ± 0.35	0.5 ± 0.16	-
Total Identified (%)				99.5 ± 0.10	100.0 ± 0.00	100.0 ± 0.00

^a^ Compounds present with percentage ≥0.1% in at least one of the *Helichrysum* spp. Data are reported as mean values (n = 3 ± SD); l.r.i^exp^: linear retention time experimentally determined on HP-5MS capillary column; l.r.i^lit^: linear retention time reported by Adams 2007 [14], NIST 14 [15], and NIST Chemistry WebBook [16].

**Table 2:** Complete chemical composition and hydrodistillation yield of essential oils obtained from dried aerial parts of three analyzed *Helichrysum* samples.

Compounds ^a^	Class	l.r.i^exp^	l.r.i^lit^	Relative Abundance (%) ± SD		
				*H. decorum*	*H. lepidissimum*	*H. umbraculigerum*
α-pinene	mh	939	935	-	2.3 ± 0.65	-
limonene	mh	1029	1031	-	-	0.4 ± 0.07
eucalyptol	om	1031	1032	-	0.2 ± 0.10	5.5 ± 1.22
γ-terpinene	mh	1060	1062	-	-	0.2 ± 0.04
terpinolene	mh	1089	1088	-	-	0.2 ± 0.05
linalool	om	1097	1089	-	-	0.2 ± 0.01
nonanal	nt	1101	1102	0.4 ± 0.06	-	-
borneol	om	1169	1168	-	-	0.5 ± 0.10
4-terpineol	om	1177	1179	-	-	0.2 ± 0.01
α-terpineol	om	1189	1189	-	-	0.3 ± 0.01
decanal	nt	1202	1205	0.5 ± 0.03	-	-
dihydroedulan IA	ac	1294	1293	-	-	0.2 ± 0.02
p-menth-1-en-9-ol	om	1295	1295 *	-	-	0.2 ± 0.03
α-copaene	sh	1377	1377	-	0.2 ± 0.06	-
β-elemene	sh	1391	1375	0.5 ± 0.04	-	-
*n*-tetradecane	nt	1400	1400 *	0.1 ± 0.19	-	-
italicene	sh	1404	1403	-	0.6 ± 0.06	-
dihydro- γ-ionone	ac	1407	1417	0.8 ± 0.07	-	-
*cis-*α-bergamotene	sh	1413	1415	-	0.2 ± 0.00	-
sesquithujene	sh	1414	1417	-	0.3 ± 0.00	-
β-caryophyllene	sh	1419	1420	8.4 ± 0.45	-	-
*trans*-α-bergamotene	sh	1435	1431	0.2 ± 0.12	-	-
α-guaiene	sh	1440	1438	0.4 ± 0.02	-	-
aromadendrene	sh	1441	1444	-	-	0.3 ± 0.03
α-humulene	sh	1455	1455	-	-	3.1 ± 0.13
dihydropseudoionone	ac	1456	1460	1.4 ± 0.12	-	-
*(E)-*β-Famesene	sh	1457	1459	1.4 ± 0.11	-	-
sesquisabinene	sh	1464	1461	-	0.1 ± 0.06	-
4,5-di-*epi*-aristolochene	sh	1469	1471	-	-	0.2 ± 0.02
β-acoradiene	sh	1471	1478	-	0.5 ± 0.06	-
γ-muurolene	sh	1480	1477	-	-	1.4 ± 0.01
ar-curcumene	sh	1481	1483	-	3.6 ± 0.35	-
γ-curcumene	sh	1483	1484	-	17.4 ± 2.10	-
α-amorphene	sh	1485	1488	-	-	0.6 ± 0.02
*trans*-β-ionone	ac	1489	1485	3.8 ± 0.51		-
β-selinene	sh	1490	1486	-	-	6.2 ± 0.30
α-selinene	sh	1498	1497	-	-	9.2 ± 0.20
α-muurolene	sh	1500	1499	-	-	0.7 ± 0.00
*epi*-zonarene	sh	1501	1501	-	-	0.5 ± 0.05
isodaucene	sh	1503	1503	1.0 ± 0.16	-	-
β-bisabolene	sh	1506	1509	4.2 ± 0.13	0.7 ± 0.10	-
α-bulnesene	sh	1510	1508	1.4 ± 0.13	-	0.4 ± 0.04
sesquicineole	os	1513	1514	-	0.8 ± 0.15	-
*trans-*γ-cadinene	sh	1514	1514	-	-	0.7 ± 0.02
β-curcumene	sh	1516	1517	-	2.9 ± 0.10	-
7-*epi*-α-selinene	sh	1522	1526	-	-	0.2 ± 0.01
δ-cadinene	sh	1523	1524	-	-	2.6 ± 0.02
cubenene	sh	1533	1531	-	-	0.3 ± 0.01
*cis*-sesquisabinene hydrate	os	1544	1559	-	0.4 ± 0.06	-
italicene ether	os	1545	1540	-	0.4 ± 0.06	-
α-calacorene	sh	1546	1546	-	-	0.5 ± 0.03
elemol	os	1550	1550	1.6 ± 0.03	-	-
*(E)-*nerolidol	os	1563	1560	-	3.0 ± 0.50	-
palustrol	os	1568	1567	-	-	0.8 ± 0.01
spathulenol	os	1578	1578	-	0.2 ± 0.06	5.3 ± 0.20
caryophyllene oxide	os	1583	1583	26.7 ± 0.10	-	-
globulol	os	1585	1584	-	-	1.5 ± 0.03
isoaromadendrene epoxide	os	1589	1594	0.6 ± 0.10	-	-
*epi*-globulol	os	1590	1587 *	-	7.4 ± 1.80	-
β-copaen-4α-ol	os	1591	1596	-	0.2 ± 0.06	-
viridiflorol	os	1593	1595	-	-	10.6 ± 0.48
cubeban-11-ol	os	1595	1601	-	-	1.5 ± 0.13
rosifoliol	os	1600	1603	1.9 ± 0.04	7.2 ± 1.20	-
guaiol	os	1601	1597	0.3 ± 0.04	2.3 ± 0.40	-
humulene oxide II	os	1608	1609	-	-	0.7 ± 0.01
*epi*-cedrol	os	1612	1611	-	0.8 ± 0.06	-
humulane-1-6-dien-3-ol	os	1613	1619	1.2 ± 0.09	0.2 ± 0.00	1.6 ± 0.01
humulol	os	1614	1618	-	-	8.0 ± 0.42
1-*epi*-cubenol	os	1629	1629	-	-	6.7 ± 0.19
γ-eudesmol	os	1632	1630	1.4 ± 0.22	-	-
α-acorenol	os	1633	1630	-	2.1 ± 0.06	-
β-acorenol	os	1637	1637	-	0.6 ± 0.00	-
isospathulenol	os	1638	1640	-	-	0.5 ± 0.06
T-cadinol	os	1640	1640	-	-	2.7 ± 0.09
α-muurolol	os	1646	1645	-	-	1.0 ± 0.11
11,11-Dimethyl-4,8-dimethylenebicyclo[7.2.0]undecan-3-ol	os	1647	1646	2.8 ± 0.30	-	-
β-eudesmol	os	1651	1649	2.1 ± 0.21	0.9 ± 0.20	1.2 ± 0.03
α-eudesmol	os	1654	1652	1.8 ± 0.32	-	-
*neo*-intermedeol	os	1655	1660	-	-	11.2 ± 0.59
pogostole	os	1656	1655	1.5 ± 0.41	-	-
intermedeol	os	1668	1666	-	6.0 ± 0.35	-
14-hydroxy-9-*epi*-*(E)*-caryophyllene	os	1670	1669	-	-	1.2 ± 0.25
bulnesol	os	1672	1668	1.6 ± 0.06	1.2 ± 0.15	-
β-bisabolol	os	1675	1672 £	-	12.5 ± 0.3	-
aromadendrene epoxide II	os	1680		1.9 ± 0.04	-	0.5 ± 0.07
α-bisabolol	os	1686	1685	1.7 ± 0.10	6.4 ± 0.25	0.2 ± 0.00
*(Z,E)-*farnesol	os	1701	1700	-	1.3 ± 0.15	-
aristol-1(10)-en-9-ol	os	1704	1704	1.0 ± 0.00	-	-
pentadecanal	nt	1713	1714	6.7 ± 0.37	-	-
β-*(Z)*-santalol	os	1715	1713	0.4 ± 0.09	5.6 ± 0.70	-
*(E,E)-*farnesol	os	1755	1740	-	0.2 ± 0.00	-
xanthorrhizol	os	1753	1752	-	0.2 ± 0.06	-
tetradecanoic acid	nt	1761	1769	-	-	1.5 ± 0.13
*neo*curdione	os	1762	1761	-	0.1 ± 0.00	-
*(Z)-*9-hexadecenal	nt	1780	1800	-	0.9 ± 0.06	-
*(Z)-*7-hexadecenal	nt	1798	1798	-	0.7 ± 0.06	-
*n-*octadecane	nt	1800		0.4 ± 0.03	-	-
*(E,E)-*farnesyl acetate	os	1843	1843	-	0.4 ± 0.00	-
hexahydrofarnesylacetone	ac	1845	1845	5.5 ± 0.43	-	2.6 ± 0.18
*Z*-(13,14-Epoxy)tetradec-11-en-1-ol acetate	nt	1849	1849	0.3 ± 0.04	-	-
*(Z)-*9-Hexadecen-1-ol	nt	1863	1863	1.2 ± 0.18	-	-
pentadecanoic acid	nt	1867	1857	-	-	0.3 ± 0.07
1-hexadecanol	nt	1876	1883	-	0.3 ± 0.06	-
*(E,E)-*farnesyl acetone	ac	1919	1920	1.0 ± 0.14	-	-
cembrene	dh	1939	1929	0.4 ± 0.15	-	-
phytol	od	1945		0.3 ± 0.01	-	0.5 ± 0.10
*m-*camphorene	dh	1952	1960	-	0.2 ± 0.06	-
cembrene A	dh	1959	1960	0.3 ± 0.39	-	-
hexadecanoic acid	nt	1963	1962	-	-	0.4 ± 0.05
geranyl linalool	od	2034	2034	-	5.6 ± 0.55	-
*n*-tetracosane	nt			0.5 ± 0.68	-	-
*n*-pentacosane	nt			2.9 ± 0.44	-	-
EO hydrodistillation yield (% *w/w*)				-	0.6 ± 0.01	-
Chemical classes				*H. decorum*	*H. lepidissimum*	*H. umbraculigerum*
Monoterpene hydrocarbons (mh)				-	2.3 ± 0.65	0.8 ± 0.12
Oxygenated monoterpenes (om)				-	0.2 ± 0.10	6.9 ± 1.44
Sesquiterpenes hydrocarbons (sh)				17.5 ± 1.26	26.5 ± 1.90	26.9 ± 0.52
Oxygenated sesquiterpenes (os)				46.7 ± 0.49	60.4 ± 1.30	55.3 ± 2.26
Diterpenes hydrocarbons (dh)				0.7 ± 0.24	0.2 ± 0.06	-
Oxygenated diterpenes (od)				0.3 ± 0.01	5.6 ± 0.55	0.5 ± 0.10
Apocarotenoids (ac)				12.5 ± 0.13	-	2.80 ± 0.15
Other non-terpene derivatives (nt)				13.0 ± 0.20	1.9 ± 0.06	2.2 ± 0.10
Total identified (%)				92.5 ± 0.84	97.1 ± 0.44	95.3 ± 0.80

^a^ Compounds present with percentage ≥0.1% in at least one of *Helichrysum* spp. Data are reported as mean values (n = 3 ± SD); l.r.i^exp^: linear retention time experimentally determined on HP-5MS capillary column; l.r.i^lit^: linear retention time reported by Adams 2007 [14], NIST 14 [15], and NIST Chemistry WebBook; * linear retention time in pubchem (www.pubchem.ncbi.nlm.nih.gov (accessed on 25 September 2021)); £: linear retention time in chemspider (www.chemspider.com (accessed on 25 September 2021)).

**Table 3:** Botanical description of the three analyzed South African *Helichrysum* species.

Species	Photo	Botanical Characters
*H. decorum DC*	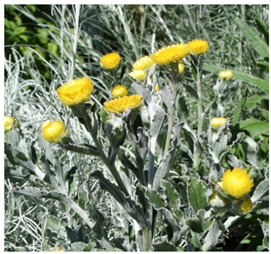	**Voucher**: HMGBH.e/9006.2020.002 Biennial or perennial herb up to 1.3 m tall grows in rough grassland or scrub, often on forest margins or in damp gullies and along streambanks. Stem stout: usually simple thinly greyish-white woolly, leafy. Radical leaves rosetted in the first year of growth, wanting at flowering, elliptic, narrowed to a broad clasping base, apex obtuse or subacute, apiculate, both surfaces thinly greyish-white woolly. Cauline leaves diminishing in size upwards, oblong-lanceolate or elliptic-lanceolate, apex usually acute, base clasping, upper surface glandular-setose, thinly cobwebby, lower thinly greyish-white woolly. Heads heterogamous, depressed-globose, across the radiating bracts, many in a large corymbose panicle. Involucral bracts, graded, imbricate, much exceeding the flowers, acute, glossy, bright yellow, radiating. Flowers c. 900–1 260, 35–110 female, 850–1,150 homogamous, yellow.
*H. lepidissimum* S. Moore	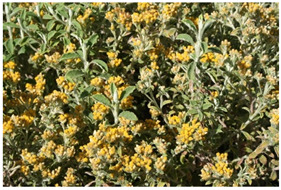	**Voucher**: HMGBH.e/9006.2017.004 Highly ramified perennial shrub grows in rocky places, particularly rock mountain tops and cliff. Leaves usually broadly elliptic lightly and loosely greyish-white woollyabove, wool glabrescent to reveal long coarse shaggy hairs, greyish-white woolly-felted below Heads homogamous campanulate, few to many in corymbos clusters terminating the branchlets. Involucral bracts, imbricate, crisped, glossy, white, creamy, or pale straw-colored. Receptacle with fibrils at least equaling ovaries Flowers 10–30, yellow
*H. umbraculigerum* Less.	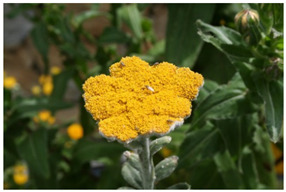	**Voucher**: HMGBH.e/9006.2020.001 Tufted Perennial Herb grows in rough grassland or scrub. Stems decumbent and rooting then erect, young parts thinly grey-woolly, leafy. Leaves very variable in shape, ranging from linear-lanceolate to elliptic and tapering at both ends; upper surface with stout or delicate glandular hairs, lower surface often thickly greyish white woolly. Heads homogamous, cylindric, many crowded and webbed together, umbrella-like disc Involucral bracts biseriate, not radiating, pellucid, canary-yellow, outer often golden-brow Receptacle nearly smooth Flowers 3–4 (6), yellow [26]

## Data Availability

Data are contained within the article.

## Sample Availability

Samples of the compounds are not available from the authors.
